# Insights into the Multilevel Structural Characterization and Adsorption Mechanism of *Sinogastromyzon* *szechuanensis* Sucker on the Rough Surface

**DOI:** 10.3390/life11090952

**Published:** 2021-09-11

**Authors:** Qian Cong, Jin Xu, Jiaxiang Fan, Tingkun Chen, Shaofeng Ru

**Affiliations:** 1 State Key Laboratory of Automotive Simulation and Control, Jilin University, Changchun 130022, China; congqian@jlu.edu.cn; 2 Key Laboratory of Bionic Engineering, Ministry of Education, Jilin University, No. 5988 Renmin Street, Changchun 130022, China; 3 College of Biological and Agricultural Engineering, Jilin University, Changchun 130022, China; xujin18@mails.jlu.edu.cn (J.X.); fanjx17@mails.jlu.edu.cn (J.F.); 4 Mechanical and Electrical College, Hainan University, Haikou 570228, China; 992948@hainanu.edu.cn

**Keywords:** *Sinogastromyzon* *szechuanensis*, adsorption, sucker, multilevel structure, mechanism analysis, spectral function

## Abstract

The present study investigates the adsorption performance and adsorption mechanism of *Sinogastromyzon szechuanensis* on different rough surfaces. The different positions of the sucker surface of *Sinogastromyzon* *szechuanensis* were observed by adopting the stereomicroscope and SEM. The observed results showed that the sucker of *Sinogastromyzon* *szechuanensis* had a multilevel structure of villi and groove. The anterior and posterior of *Sinogastromyzon* *szechuanensis* had different microscopic morphologies. The surface roughness of the adsorption substrate ranged from 7 μm to 188 μm. Adsorption strength of *Sinogastromyzon* *szechuanensis* and the conventional sucker on different rough surfaces were measured by a purposely designed device. The results showed that the back of *Sinogastromyzon* *szechuanensis* mainly provided the adsorption strength. The adsorption strength of the conventional sucker gradually decreased with surface roughness increasing, but the adsorption strength of *Sinogastromyzon* *szechuanensis* had not changed significantly. Based on the experimental results, the adsorption mechanism of *Sinogastromyzon* *szechuanensis* on the surface with different roughness was analyzed by the spectral function. The *Sinogastromyzon* *szechuanensis* sucker with a multilevel structure worked well on the rough surface, which led to *Sinogastromyzon* *szechuanensis* with a good sealing on the rough surface. The present work could help to develop a new type of sucker with effective adsorption performance on a rough surface to meet the needs of the engineering field.

## 1. Introduction

With the continuous development of science and technology, automatic machinery gradually popularizes in engineering. As the basic component of the automatic adsorption device, the suction disc has rapidly expanded its application in the field of industrial automation. The developed equipment with adsorption performance can meet the needs of some engineering fields and increase operational efficiency and protect human safety and health from hazardous tasks, such as robots with wall-climbing functions and unmanned aerial vehicles with static adsorption on the wall [1,2,3,4]. However, it is well known that the surface morphology of the substrate has a great influence on the adsorption properties of the sucker [5,6,7,8]. Therefore, purposes such as these have promoted researchers and engineers to develop new methods to improve the adsorption performance of the sucker or design new kinds of suction discs, whether from materials, structures, or other means. Many living creatures possess extremely strong skills to adapt to the environment and survive to date with evolution in nature for millions of years. One typical phenomenon is that the organisms have formed the capability to stick to various surfaces under different living conditions and weather. It has aroused great attention from researchers and engineers to improve or develop new kinds of suction or machinery with high adsorption performance. The adsorbed organisms could be divided into two types according to the living environment: the terrestrial adsorption organism and the aquatic organism, such as gecko, tree frog, leech, abalone, octopus, and so on [9,10,11,12,13].

Many studies have been carried out on the terrestrial adsorption organism to develop the bionic sucker with remarkable performance and optimize the existing suction disc. The gecko can freely climb on the horizontal or vertical walls, owing to the van der Waals force between the sole and the solid caused by the special structure of the sole [14,15,16,17]. The adsorption mechanism exists in organisms, for example, the spider [18,19,20]. Meanwhile, many organisms adhere to and climb on the wall by the liquid tension and capillary force between the body surface and the solid, such as the tree frog, fly, beetle, ant, and so on [21,22,23,24,25]. In short, the terrestrial organisms with adsorption properties can attach to the solid surface owing to originating from several forces, i.e., the van der Waals force, capillary force, viscous force, mechanical interlocking, and so on [12,26,27].

Many studies have been carried out on the adsorption mechanism to the terrestrial organism, but there are many impurities between the adsorption interface and the attached substrate, such as water and oil stains, which will affect the contact interfaces and weaken many forms of adsorption forces [27,28,29]. Conditions such as these are similar to the aquatic adsorption organisms. The number of suckers can be classified into two categories: multiple suckers, living things represented by octopus and leech, and single suckers, living things represented by abalone and remora. The surface of the octopus suction cup with a gully shape has a convex body that can squeeze the inner cavity of the suction cup to form a vacuum so that the octopus can adhere to different surfaces [30,31]. Meanwhile, the hairy structure on the convex body surface can also seal the adsorption of octopus, which would be helpful to the adsorption process [31]. The leech forms a vacuum during the adsorption process, which is not easy to fall off due to the surface of the leech sucker with a gully-like structure [11]. Because the surface of the abalone sucker has a hairy structure that can facilitate the formation of vacuum adsorption, it can be well sealed on the rough surface, and three kinds of forces that affect the adsorption performance of abalone are analyzed. A quantitative calculation method of the abalone total adsorption force is proposed [32,33]. Meanwhile, it is found that the suckers of the northern clingfish and *Petromyzon marinus* have a hairy structure, and this structure is conducive to adsorption on rough surfaces [34,35,36]. Shortly, underwater adsorption of organisms adheres to various substrates under the single action or interaction of vacuum-adsorption force, van der Walls force, capillary force, mechanical interlocking, and so on.

The adsorption of the aquatic organisms is related to vacuum adsorption caused by the hairy structure. The published papers show that the suckers with hairy structures have better adsorption performance on rough surfaces than the conventional sucker [11,26,34,36,37,38]. The mechanism of the influence of the hairy structure on the aquatic organism adsorption on the rough surface has not been quantitatively analyzed. Moreover, it is well known that the living environment and adsorbed objects of the different organisms are different, but the structure and adsorption mechanism of each adsorption organism also have their characteristics. However, the adsorption structure and mechanism of the organisms have similarities and regularity to reach adsorption stability.

The *Sinogastromyzon* belongs to the *Balitoridae*, which is one unique kind of fish in China and Vietnam [33]. In addition, the *Sinogastromyzon* has evolved a suction cup that can stably adsorb on the rough rock surface to resist the rapid water flow. Moreover, there are few studies on the quantitative analysis of the adsorption mechanism of *Sinogastromyzon* on the rough surface. Therefore, the present work takes the *Sinogastromyzon* *szechuanensis* as the object to observe the surface microstructure of the sucker, and the adsorption performance of *Sinogastromyzon* *szechuanensis* on the fabricated surfaces with different roughness are tested. In addition, the effects of the different positions of the *Sinogastromyzon* *szechuanensis* on its adsorption performance were analyzed. Combined with adopting the mathematical method to characterize the adsorption surface roughness, the influence mechanism of the micro multistage structure of the sucker on the adsorption performance of *Sinogastromyzon* *szechuanensis* would be quantitively analyzed. The study will be helpful to optimize and develop the sucker with high adsorption performance and satisfy the application requirements of some special engineering fields, such as the wall-climbing robot for welding, maintenance, detection, and other purposes, and the unmanned aerial vehicle with adsorption for military reconnaissance.

## 2. Materials and Methods

### 2.1. Experimental Specimens

The *Sinogastromyzon* *szechuanensis* species employed in the experiment are not nationally protected or scarce, and the specimens were purchased from Shanghai Xiling Biological Technology Co., Ltd. (Shanghai, China).

The study was carried out in the Key Laboratory of Engineering Bionics, Ministry of Education, Jilin University (SYXK (Jilin) Key Laboratory of Engineering Bionics, Ministry of Education, Jilin University). It was carried out under the supervision of the Institutional Animal Care and Use Committee of Jilin University (IACUC), and complied with the requirements of Jilin University and China for the ethical welfare of laboratory animals. The breeding conditions were carried out by GB/T 35823-2018 [39] standard, and the freshwater conforming to GB 5749-2006 [40] standard was used for breeding. All specimens were cultivated in the fish container (500 × 500 × 500 mm^3^ in size) with the thermostatic device. Moreover, the laboratory animals were fed regularly.

The specimen was euthanized by immersing it in ethanol solution during the study. The *Sinogastromyzon* *szechuanensis* was taken out for observation and adsorption test until the gills stopped moving. The volume concentration of the ethanol solution was 25 mL/L. The *Sinogastromyzon* *szechuanensis* carcasses were treated in a centralized and harmless manner after the test.

### 2.2. Structural Characterization

#### 2.2.1. Stereo Microscopy

**Figure 1:**
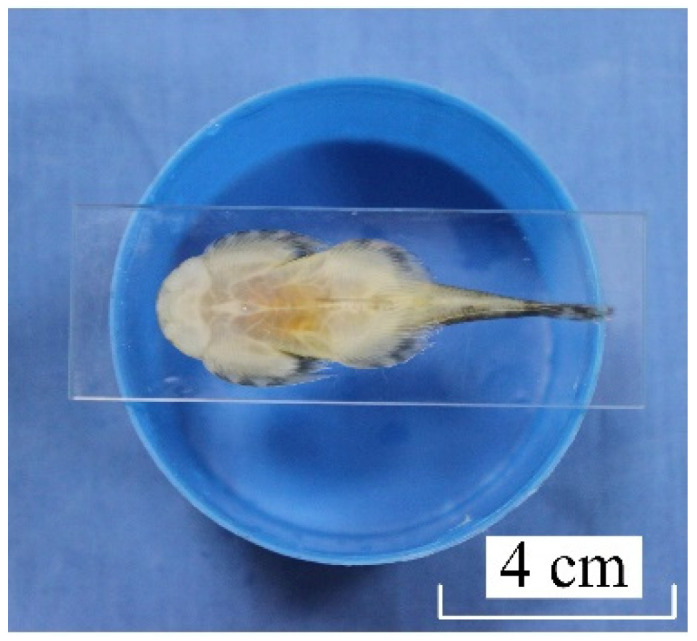
*Sinogastromyzonszechuanensis* adsorbed on the glass slide.

The morphology of the adsorption side of the *Sinogastromyzon* *szechuanensis* was observed by stereo microscopy (Zeiss Stemi 2000-C, Oberkochen, Germany) to get a preliminary understanding of the structure of *Sinogastromyzon* *szechuanensis*. The non-dehydrated sample was quickly and smoothly placed on the glass slide to prevent the biological structure from changing after being euthanized. The good adsorption between the specimen and the glass slide would be formed owing to the gravity and adsorption of the *Sinogastromyzon* *szechuanensis*. The slide with the sample was inverted and placed on the cylinder with color to observe the morphology of the adsorption side. The color of the cylinder was blue during the test, as shown in Figure 1. The glass slide and the cylinder were placed under the stereo microscope to observe the surface morphology of the adsorption side of the *Sinogastromyzon* *szechuanensis*, which would lay the foundation for SEM observation.

#### 2.2.2. Scanning Electron Microscopy

Combined with the results of the stereomicroscope, the SEM observation area of the *Sinogastromyzon* *szechuanensis* was determined. Samples for scanning electron microscopy examination were carefully divided into small pieces. The samples were placed into a glutaraldehyde solution with a mass solution of 2.0% for 3 h at 3 °C to keep the original morphology. The samples were rinsed three times by using the phosphate buffer saline, and each time lasted 15 min. The samples were then dehydrated by the ethanol solution with different mass concentrations of 50%, 70%, 80%, 90%, and 100%. Except for immersing three times in ethanol absolute, the samples were immersed in the ethanol solutions of other concentrations for one time, and each time lasted for 10 min. Finally, the samples dried in the vacuum freeze-drying box for 48 h were treated with gold spray. The surface morphology of the adsorption side of the *Sinogastromyzon* *szechuanensis* was observed by scanning electron microscopy.

### 2.3. Fabrication and Surface Characterization of Substrates

The adsorption substrate with roughness was fabricated by the mold method to test the adsorption performance of *Sinogastromyzon* *szechuanensis* on different rough surfaces.

**Figure 2:**
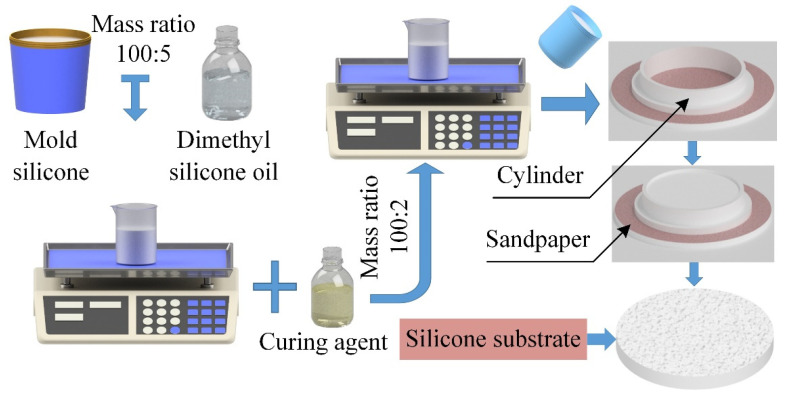
The fabricated procession of the adsorption substrate with a rough surface.

As shown in Figure 2, the fabricated procession of the adsorption substrate with roughness was divided into three steps: (1) Mold preparation: In the present study, sandpaper was used to simulate the surface roughness of cobblestone in the living environment of *Sinogastromyzon* *szechuanensis.* The sandpaper with mesh numbers of 40#, 80#, 120#, 240#, and 320# were regarded as the base, respectively. A cylinder with a diameter of 70 mm and a depth of 10 mm was placed above the sandpaper. A thin layer of Vaseline would be evenly applied on the surface of the sandpaper and the inner wall of the cylinder. (2) Softening of the mold silicone: Dimethyl silicone oil was added to the mold silicone to achieve a better reproduction of the roughness of the sandpaper surface. The mass ratio of the dimethyl silicone oil to mold silicone was 5:100, and the mold silicone mixture was stirred for 3 min to fully soften the silicone and increase the fluidity of the mixture. Then, the mold silicone mixture was allowed to stand for 5 min. (3) Curing of the mold silicone mixture: The curing agent was added to the mold silicone mixture at a mass mixing ratio of 2:100, and the mixture was stirred evenly. The final prepared mixture was poured into the cylinder, and it was allowed to stand at ambient temperature (18 °C) for 2 h. The adsorption substrate with a rough surface was removed after curing.

**Figure 3:**
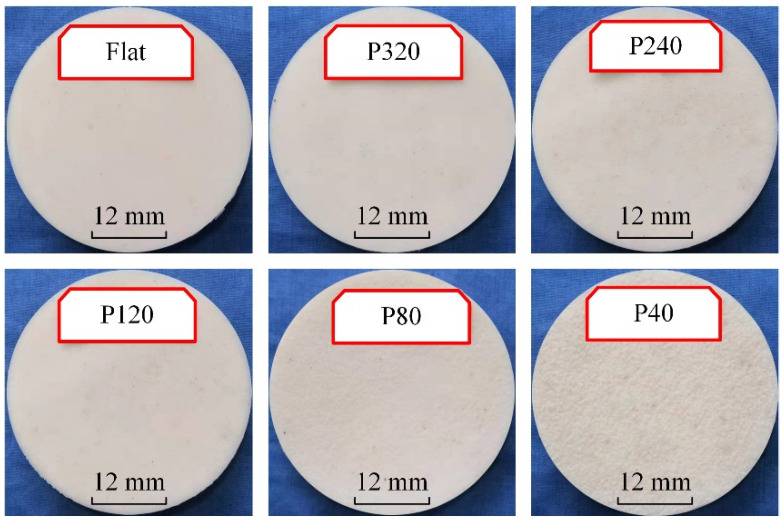
Adsorption substrates with rough surfaces.

Meanwhile, the sandpaper was replaced with the normal glass, and the same process fabricated the adsorption substrate with a smooth surface. The prepared samples with different roughness were as shown in Figure 3.

**Figure 4:**
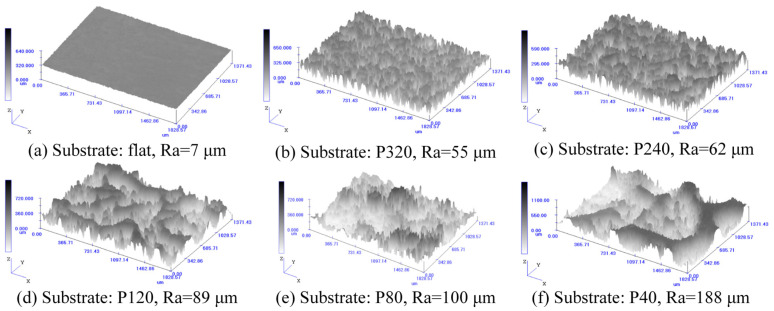
The roughness of the adsorption substrate surface.

Although the surface roughness of the sandpaper increases with the decrease in the mesh number of the sandpaper, the roughness is not represented by using the mesh number of the sandpaper. Moreover, the surface roughness of the adsorption substrate fabricated by the mold method might not always be the same as that of the sandpaper template. The surface roughness of the silicone plate was accurately measured by using 2EXT (Olympus Corporation, Tokyo, Japan). Meanwhile, the morphology of the rough surface of the adsorption substrate could also be visually observed, as shown in Figure 4.

### 2.4. Adsorption Capability Measurement

**Figure 5:**
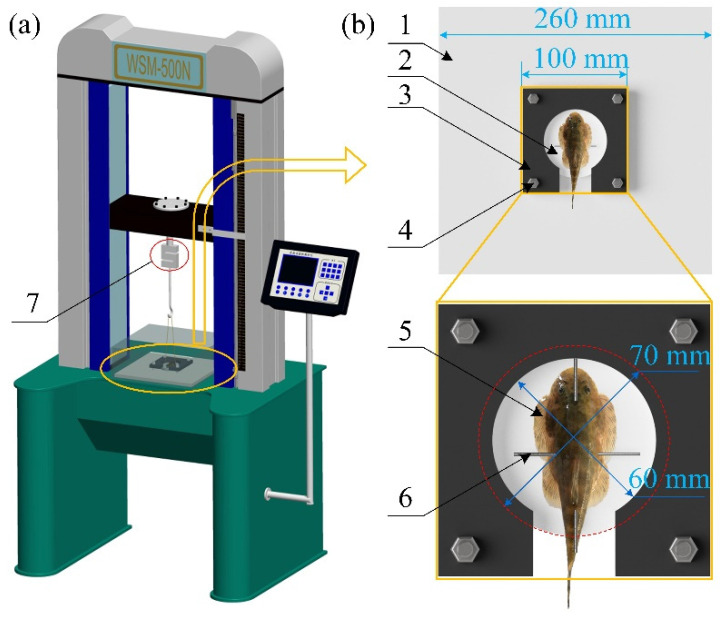
Adsorption force test device: (**a**) a pulling machine to measure the adsorption force of *Sinogastromyzon szechuanensis*; (**b**) the specimen placed diagram. 1. Base; 2. silicone substrate with a rough surface; 3. square aluminum alloy plate; 4. bolts; 5. sample; 6. steel needle; and 7. force sensor.

The adsorption strength was measured using a pulling machine to evaluate the effect of the surface roughness on the adsorption performance of the *Sinogastromyzon szechuanensis*, as shown in Figure 5a. A tool for testing the *Sinogastromyzon* *szechuanensis* on the silicone substrate with a rough surface was purposely built to prevent the adsorption substrate from being pulled up or slipping during the test.

As shown in Figure 5b, the base, silicone substrate, and square aluminum alloy plate were connected using bolts to ensure that the silicone base and adjacent components always adhered together during the test. The replacement of the silicone base could be realized by loosening the bolts.

Because there was mucus on the back of the *Sinogastromyzon* *szechuanensis*, it could not be directly connected to the force sensor. Therefore, the steel needles were inserted into the *Sinogastromyzon* *szechuanensis* from the vertical and parallel directions to the body axis (in agreement with Chuang et al. [41]). The steel needle inserted horizontally was placed above the steel needle inserted vertically, as shown in Figure 5b. The hooks were connected with the steel needle placed horizontally, and the force sensor was linked with hooks through a nylon rope during the test. The PC controlled the pulling machine during the test, and the force sensor could be moved up and down at a speed of 200 mm/min to remove the *Sinogastromyzon* *szechuanensis* from the substrate. Simultaneously, the maximum adsorption force was measured and recorded during the movement of the force sensor. The precision of the force sensor was 0.01 N, which met the requirement of the test. The adsorption strength was calculated using the Equation *P* = *F*/*S*, where *P* is the pressure, *F* is the adsorption force, and *S* is the adsorption area of the *Sinogastromyzon* *szechuanensis* adsorption on the smooth glass surface determined by using reverse technology before the experiment. Owing to the specimens adopted in the test with different lengths, the adsorption area of *Sinogastromyzon* *szechuanensis* on the substrate would also be different.

## 3. Results

### 3.1. Macrostructural Characterization

**Figure 6:**
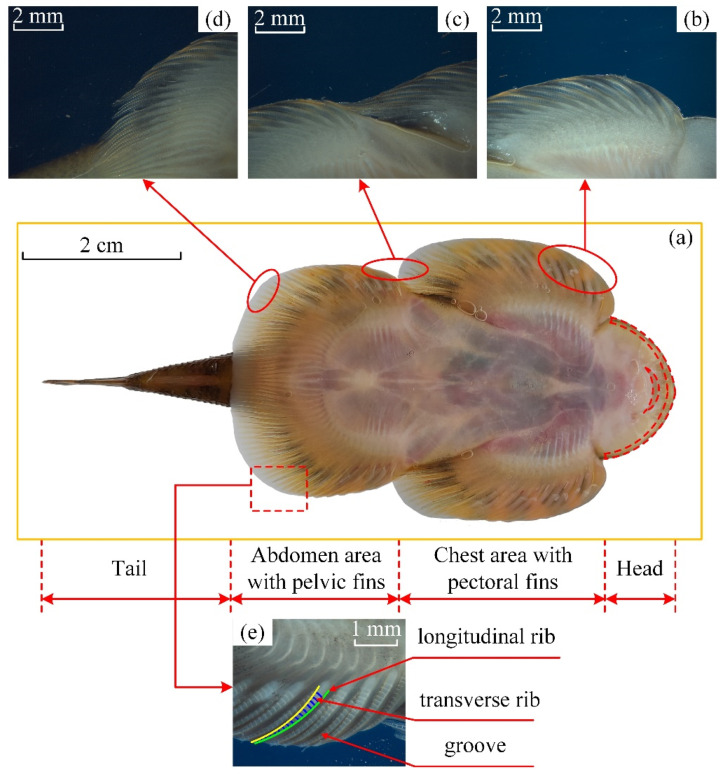
Macrostructural morphology of the adsorption side of *Sinogastromyzon* *szechuanensis*, observed with stereomicroscope: (**a**) photograph shown the pelvic view of the *Sinogastromyzon* *szechuanensis*; (**b**) pectoral fins; (**c**) the intersection of the pectoral fins and the pelvic fins; (**d**) the pelvic fins; and (**e**) the morphology of the fin of the *Sinogastromyzon* *szechuanensis*.

The macroscopic morphology of different parts of the adult specimen was observed by stereomicroscope. The *Sinogastromyzon* *szechuanensis* could be divided into the head, the chest area, the abdomen area, and the tail, as shown in Figure 6.

The edge of the head of the *Sinogastromyzon* *szechuanensis* was the soft and deformable tissue, which might help the *Sinogastromyzon* *szechuanensis* peel from the substrate surface. The pelvic fins of both sides of the *Sinogastromyzon* *szechuanensis* overlapped at the tail. Images a–c of Figure 6 illustrated that the fins of the different locations on the adsorption side of the *Sinogastromyzon* *szechuanensis* had similar structures. As shown in Figure 6e, the longitudinal ribs with the triangular cross-sectional shape were distributed orderly on the surface of the fins, and the grooves were formed between the adjacent purlines. The transverse ribs are regularly distributed on both sides of the longitudinal ribs. The cross ribs would enhance the strength of the fins and help *Sinogastromyzon* *szechuanensis* live in the river with rapid speed.

### 3.2. Microstructural Characterization

**Figure 7:**
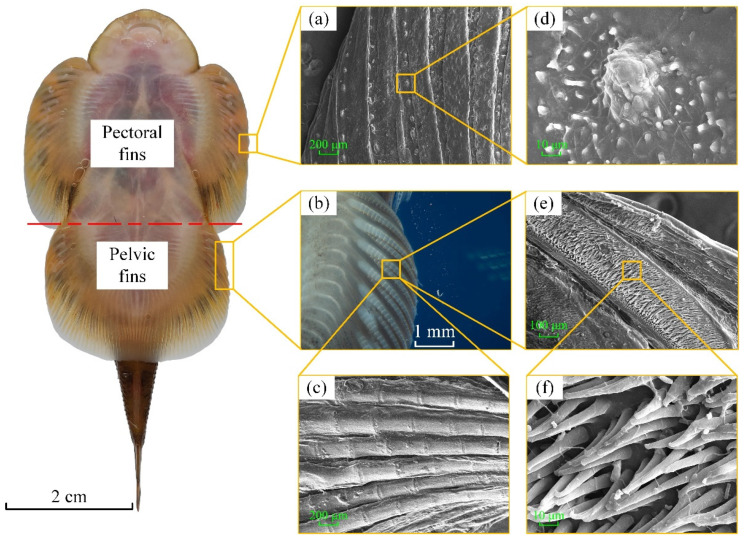
Microstructural characterization of the *Sinogastromyzon* *szechuanensis*, observed with SEM: (**a**) the papillae distributed on the longitudinal ribs of the pectoral fins; (**b**) the morphology of the pelvic fins; (**c**) a layer of densely distributed trichomes on the surface of the pelvic fin; (**d**) the magnification of the papillae in (**a**); (**e**) the trichome covered the longitudinal ribs surface of the pelvic fins; (**f**) the magnification of the trichome in (**e**).

The microstructure was observed to determine whether the pectoral fins and pelvic fins of the *Sinogastromyzon* *szechuanensis* have similar strip-like macrostructures. As shown in Figure 7a,d, the papillae regularly distributed on each longitudinal rib of the pectoral fins, the number was about 12 to 18. The papillae had a conical shape with a bottom diameter of about 30–40 μm and a spacing of about 150–200 μm. The smaller size transverse ribs were found on both sides of the longitudinal ribs of the pelvic fins of the *Sinogastromyzon* *szechuanensis* by stereoscopic microscopy, as shown in Figure 7b. Therefore, the microstructure of the pelvic fins was observed by SEM, and the longitudinal ribs’ surface of the pelvic fins area was covered by the trichome, as shown in Figure 7e,f. Additionally, this was different from the longitudinal ribs located in the pectoral fins. It could be seen from Figure 7f that the height of the trichome was about 50 μm and the diameter of the trichome increased from bottom to top. The top diameter of the trichome was about 2~5 μm. In the comparison of Figure 7d,f, the distribution density of the trichome was higher than that of the papillae.

The gently scraped fins were observed by SEM to verify whether the sucker of the *Sinogastromyzon* *szechuanensis* had a multilevel structure to adsorb on different surfaces. During the scraping process, the stereomicroscope was regarded as the magnifier to obtain a good processing effect. As shown in Figure 7c, the surface of the treated fin still had the transverse ribs with a length and width of approximately 200 μm and 100 μm, respectively. The distribution of transverse ribs on the surface of longitudinal ribs was similar to that of bamboo canes. Comparing Figure 7c,f, it can be determined that there was a layer of densely distributed trichomes on the surface of the pelvic fin. Combining Figure 7c,d with Figure 7f, it can be seen that the surfaces of the pectoral fin and pelvic fin had groove-like ribs, and the rib surfaces had different microstructures. The secondary structure on the pelvic fin surface was composed of ribs and a densely distributed trichomes layer.

### 3.3. Adsorption Strength

#### 3.3.1. Mechanical Properties of the *Sinogastromyzon* *Szechuanensis*

**Figure 8:**
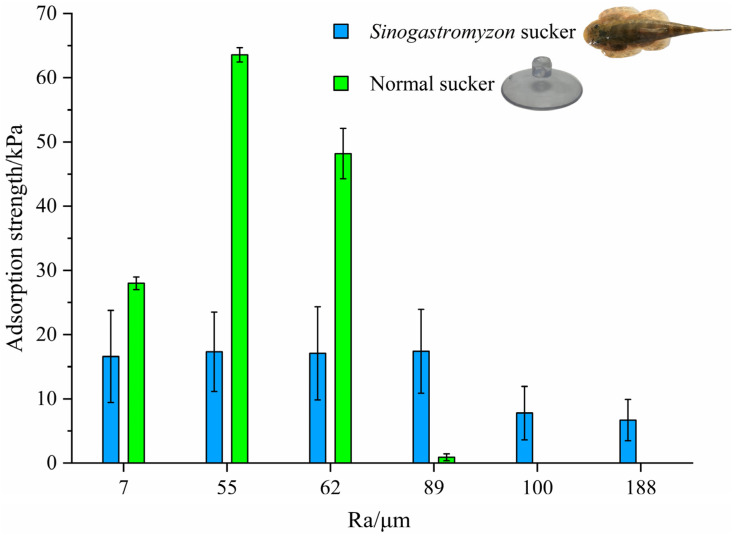
The adsorption strength of *Sinogastromyzon* *szechuanensis* on different rough surfaces. Notice: The adsorption strength of the normal sucker on the surface with roughness of 100 μm and 188 μm was 0 kPa.

Nine adult samples were tested six times on each rough substrate during the test. The adsorption strength of each sample on the rough surface was calculated by combining the adsorption area measured by reverse engineering. The adsorption area of the *Sinogastromyzon* *szechuanensis* on the substrate was from 259 mm^2^ to 432 mm^2^. In addition, the adsorption force of the two normal suckers (made by PVC, one kind of artificial sucker) with a diameter of 25 mm on each rough surface was also tested and served as a reference for evaluating the performance of the *Sinogastromyzon* *szechuanensis*. The average results were taken as the adsorption strength of the sample in question. Figure 8 summarizes the experimental results.

As shown in Figure 8, when surface roughness was less than 62 μm, the adsorption strength of the conventional sucker on the smooth surface was better than that of the *Sinogastromyzon* *szechuanensis*, and the conventional sucker had the max adsorption strength on a substrate with a surface roughness of 55 μm. The max valve of the normal sucker on the rough surface was 63.56 kPa. When the adsorption substrate surface’s roughness was 89 μm and 100 μm, respectively, the adsorption strengths of the conventional sucker and the *Sinogastromyzon* *szechuanensis* would significantly reduce. Moreover, as the roughness increases, the adsorption strength of the conventional sucker gradually increased and then decreased until it could not adsorb on surfaces with a roughness greater than 100 μm. It illustrated that the *Sinogastromyzon* *szechuanensis* could maintain stable adsorption performance on rough surfaces, and the surface roughness of the substrate had a significant effect on the adsorption performance of the normal sucker.

#### 3.3.2. Experiment on the Influence of the Fin on Adsorption Strength

**Figure 9:**
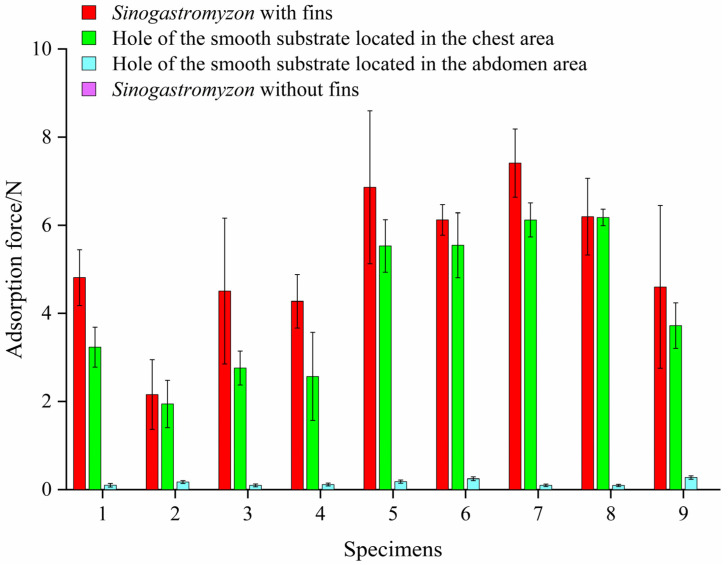
Influence of different locations of *Sinogastromyzon* *szechuanensis* on the adsorption force. Notice: Force of the *Sinogastromyzon* *szechuanensis* without fins on the smooth substrate was 0 N.

Owing to the microstructure difference between the pectoral fins and the pelvic fins, the role during the adoption process might be different. This was very interesting. Meanwhile, to analyze the adsorption mechanism and the role of the fin in the adsorption process, the experiment on the influence of the fins on the adsorption force was carried out. Firstly, a hole with a diameter of 3 mm was manufactured in the center of the smooth silicone substrate. Then, the adsorption force of the adult specimen without fins on the smooth substrate was measured under the same conditions. When the chest or abdomen area is adsorbed in the perforated area, this adsorption area will not form a good seal with the substrate. Therefore, it was considered that this area would not affect the formation of the adsorption force. For example, in order to analyze the role of the pelvic fin during the adsorption process, the adsorption force was measured when the hole of the smooth substrate was located in the chest area of the *Sinogastromyzon szechuanensis*. The experimental results are shown in Figure 9.

It could be found from the experimental results that the adsorption force of the specimens without fins on the substrate was 0 N. The result showed that the adsorption force of the specimen on the substrate surface was mainly caused by the vacuum formed between *Sinogastromyzon* *szechuanensis* and substrate. When the hole was located in the adsorption area between the chest of the sample and the smooth substrate, the adsorption force between the loach and the substrate was close to that of the *Sinogastromyzon* *szechuanensis* on the surface of the normal substrate. However, when the hole was located in the adsorption area between the abdomen of the specimen and the smooth substrate, the adsorption force between the specimen and the substrate was smaller, close to 0 N, as shown in Figure 9. The adsorption force generated by the abdomen region contributed more than 60.1% to the adsorption force formed by the *Sinogastromyzon* *szechuanensis* on a normal rough surface. Therefore, the influence of the fins in the abdomen area on the adsorption performance of *Sinogastromyzon* *szechuanensis* was significantly greater than that in the pectoral area.

## 4. Discussion

Based on the experiment, there was a multilevel structure composed of non-smooth morphology and microstructure layer on the surface of the sucker of the *Sinogastromyzon* *szechuanensis*. The multi-level structures make a vacuum between the *Sinogastromyzon* *szechuanensis* and the surface of the substrate. Suppose the sucker of the *Sinogastromyzon* *szechuanensis* should maintain the stable vacuum adsorption on the substrate surface. In that case, a good sealing effect must be kept between the suction cup and the substrate surface. Therefore, the influence of the multilevel structures on the sealing property of the sucker on the rough surface will be studied to analyze the influence mechanism.

### 4.1. Qualitative Discussion

**Figure 10:**
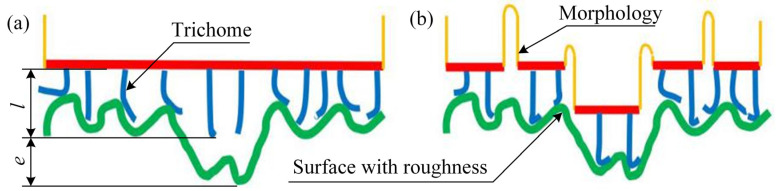
Illustration of the effect of single-stage and multistage structures on the adsorption performance of the *Sinogastromyzon* *szechuanensis*: (**a**) trichomes in a single-level structure; (**b**) trichomes in a multi-levels structure. *l* representation of the length of the trichome; *e* representation of the gap between the trichome and the surface.

It could be seen from Figure 9 that the influence of the fins located in the chest area on the adsorption force of the *Sinogastromyzon* *szechuanensis* was smaller than that of the fins situated in the abdominal area. Based on the experimental observation result, there was a significant difference in microstructure between the fin in the chest area and the abdomen area. Therefore, the influence of the microstructure of the fin in the chest area on the adsorption performance of *Sinogastromyzon* *szechuanensis* was studied. Figure 10 illustrates the mechanism of single-level structure and multilevel structures on the adsorption performance of *Sinogastromyzon* *szechuanensis*. When the surface roughness is small, the single-level structure can fill the microporous on the substrate surface and form a good sealing property between the surface and *Sinogastromyzon* *szechuanensis*. However, the single-level structure cannot sufficiently fill the microporous surface due to the roughness increase. Many organisms in nature have formed multilevel structures to adapt to their living environment [42,43], such as lotus leaf and butterfly, and each level structure has different characteristics and functions. For the multilevel structure, the first level of the multilevel structures will fill the large microporous surface on the substrate, and trichomes will fill it again. It overcomes the disadvantage that the single-level structure cannot fill the surface with large roughness. The conical appearance of the trichome is also more conducive to filling the microporous structure on the substrate surface and improving the sealing performance between the sucker and substrate surface.

It can be concluded that the trichome of the multilevel structures plays an important role in the sealing performance of the sucker, and the rough surface will also affect the adsorption property of the sucker. Although the influence mechanism of the multilevel structures on the adsorption performance of *Sinogastromyzon* *szechuanensis* has been qualitatively analyzed, the mechanism of the influence of different morphologies in the multilevel structures on the adsorption performance of the sucker on different rough surfaces has not been studied. In the present study, combined with the observed microstructure of the sucker of the *Sinogastromyzon*, the influence mechanism of the multilevel structures on the adsorption property of the sucker on the substrate with roughness is explored by using the mathematical method.

### 4.2. Quantitative Discussion

The rough surface of the substrate consists of many peaks and troughs according to the definition of surface roughness. Therefore, the surface with roughness is simplified during the study, and it is considered that the surface roughness is a periodic wave with amplitude. The spectral function in mathematics can describe the rough surface, and the published references [44,45] have taken the spectral function c(q) to characterize the rough surface. The calculated process was reported in detail [44,45]. Therefore, compared with the mechanism that analyzes the trichome as a single-stage structure on the adsorption performance of the *Sinogastromyzon* by Chuang et al. [41], the study takes the spectral function to quantitatively analyze the influence mechanism of the multilevel microstructure on the adsorption performance of the *Sinogastromyzon* *szechuanensis*.

The *Sinogastromyzon* *szechuanensis* often adsorbs on the cobblestone in the living environment, and the reference has established the spectral function for the surface roughness of the cobblestone in nature [45], as described by the following expression:

(1) $$\sigma^{2}=<h^{2}>(q_{0},q_{1})=2\pi \int_{q_{0}}^{q_{1}}q\cdot c(q)dq$$

Here, σ represents the standard deviation of the surface roughness; c(q) is the spectral function of the rough surface; h is the peak amplitude of periodic wave represented by c(q); <⋅⋅⋅> stands for ensemble averaging; in the waveform used to describe the surface roughness of the substrate, $q_{0}$ is the critical frequency, and $q_{1}$ is the maximum frequency of the surface roughness. It means that the frequency of the roughness of the substrate surface in the spectral function varies from $q_{0}$ to $q_{1}$.

The logarithm of the critical frequency and the maximum frequency of the waveform describing the surface roughness of the cobblestone can be determined by Persson et al. [45]. Namely, $\log q_{1}$ and $\log q_{0}$ are approximately equal to 4.5 and 2.5, respectively. Based on the study [45], the logarithm of the spectral function c(q) used to represent the rough surface of the cobblestone is approximately linear with the logarithm of the waveform frequency. The following expression describes the relationship:

(2) logc(q)=−2−4logq

Therefore, Equation (1) can be simplified as:

(3) $$\sigma^{2}=<h^{2}>(q_{0},q_{1})=2\pi \int_{q_{0}}^{q_{1}}10^{-2}\cdot q^{-3}dq=\pi \cdot 10^{-2}\cdot {\left[q^{-2}\right]}_{q_{0}}^{q_{1}}$$

Based on the SEM observation results of the abdominal morphology of the *Sinogastromyzon szechuanensis*, the length of the transverse rib and the trichome are about 200 μm and 50 μm, respectively. Therefore, the first-level structure can fill the waveform with a width λ greater than 400 μm. The trichome of the second level will fill the waveform with a wavelength of fewer than 400 μm. The critical wave used to describe the rough surface of the multilevel structures’ adsorption substrate is a waveform with a wavelength of 400 μm. Additionally, the relationship between the frequency and the wavelength is given by Nayak and Persson et al. [44,45]:

(4) q=2π/λ

Therefore, the logarithm of the critical waveform frequency and maximum waveform frequency $\log q_{0}^{\prime }$ and $\log q_{1}^{\prime }$, used to describe the rough surface of the multistage adsorbed substrate are about 4.2 and 4.5, respectively. According to Equation (1), it can be determined that the average depth of the microporous filled by the trichomes in the multistage structure is 9.68 μm, which is less than the length of the trichomes in the abdominal fin of *Sinogastromyzon szechuanensis*. If the length of the trichome of the multilevel structure is greater than the depth of the microporous layer, the trichome will not only fill the microporous layer, but the remaining length of the trichome will be overwhelmed and fill the interface between the substrate surface and the groove structure of the *Sinogastromyzon szechuanensis*. The trichome between the sucker and the rough surface acts as a gasket, ensuring that the sucker adsorbed on the rough surface has a good seal performance.

Compared with the role played by different structures in filling rough surfaces, the trichomes of the single-stage structure would be full of the adsorption substrate surface regardless of the size of the microporous layer. Therefore, the critical frequency ($q_{0}^{\prime \prime }$) and the maximum frequency ($q_{1}^{\prime \prime }$) of the rough surface waveform of the adsorption matrix describing the single-stage structure of trichomes are equal to the critical frequency ($q_{0}$) and the maximum frequency ($q_{1}$) of the rough surface waveform of the cobblestone, respectively. Based on Equation (3), the value of the standard deviation of the roughness σ is about 560 μm. It means that if the sucker has a good sealing effect, the height of the single-level trichomes should be greater than 560 μm. The filling depth of the trichomes in the multilevel structure on the rough surface of the adsorbed substrate is smaller than that of the single-level structure on the rough surface of the substrate. Compared with the height of the trichome determined by the micro observation experiment of *Sinogastromyzon szechuanensis*, the trichome of the single-stage cannot fill the rough cobblestone. Moreover, this will affect the adsorption performance.

In short, on the rough cobblestone surface, the trichomes in the multilevel structure can form a better seal with a rough surface than the single-level trichome.

## 5. Conclusions

The fins in the chest area and the abdomen area of *Sinogastromyzon* *szechuanensis* have a multilevel structure, but their microstructure is different. The microstructure of the fin in the chest area is regularly distributed papillae, and the microstructure of the fin in the abdomen area is a densely distributed trichome. Based on the adsorption force test, the sucker with the multistage structure on the rough surface is more stable than that of the normal sucker. The adsorption force of the abdomen area of the *Sinogastromyzon szechuanensis* on the rough surface is approximately equal to that of the whole *Sinogastromyzon* *szechuanensis* on the rough surface, and is greater than that of the chest area of the *Sinogastromyzon* *szechuanensis* on the rough surface.

In the study, based on the observation of the microstructure of the fin of the *Sinogastromyzon* *szechuanensis*, the mechanism of the influence of the multilevel structure on the adsorption performance is qualitatively analyzed, and the sealing mechanism of the sucker with the multilevel structure on the rough surface is quantitatively analyzed by using the spectrum function.

Based on the experiment results, this paper will provide a reference for investigating the influence mechanism of the multistage structure on the adsorption performance by adopting the mathematical method. In addition, this finding may help researchers and engineers to optimize the structure of the existing sucker and develop the novel sucker with high adsorption performance, which will be highly useful within the designing wall-climbing robot, unmanned aerial vehicles, and so on.

## Data Availability

The data supporting the findings of this study are available in the article.
